# An *in vitro* platform for study of the human gut microbiome under an oxygen gradient

**DOI:** 10.1007/s10544-023-00653-3

**Published:** 2023-04-04

**Authors:** James Comolli, David I. Walsh, Johanna Bobrow, Chelsea L. Lennartz, Nicholas J. Guido, Todd Thorsen

**Affiliations:** Biological & Chemical Technologies Group, MIT Lincoln Laboratory, Lexington, MA USA

**Keywords:** Microbiome, Gut, *In vitro* model, Oxygen, Microbial community

## Abstract

The complex, dynamic environment of the human lower gastrointestinal tract is colonized by hundreds of bacterial species that impact health and performance. Ex vivo study of the functional interactions between microbial community members in conditions representative of those in the gut is an ongoing challenge. We have developed an *in vitro* 40-plex platform that provides an oxygen gradient to support simultaneous maintenance of microaerobic and anaerobic microbes from the gut microbiome that can aid in rapid characterization of microbial interactions and direct comparison of individual microbiome samples. In this report, we demonstrate that the platform more closely maintained the microbial diversity and composition of human donor fecal microbiome samples than strict anaerobic conditions. The oxygen gradient established in the platform allowed the stratification and subsequent sampling of diverse microbial subpopulations that colonize microaerobic and anaerobic micro-environments. With the ability to run forty samples in parallel, the platform has the potential to be used as a rapid screening tool to understand how the gut microbiome responds to environmental perturbations such as toxic compound exposure, dietary changes, or pharmaceutical treatments.

**Supplementary Information:**

The online version contains supplementary material available at 10.1007/s10544-023-00653-3.

## Introduction

The microbiome colonizing the human gut consists of trillions of microbes that interact with the body to perform integral functions that maintain health and, when perturbed, affect the progression of disease (Huttenhower et al. 2012). The colonic microbial community composition in adults is relatively similar at the higher taxonomic levels but, at finer resolution, the microbial species and strains vary individually in identity and abundance (Mehta et al. 2018). Culture, location, age, and health status play major roles in determining the baseline microbial composition, while diet can have both short and long-term impacts on diversity (David et al. 2014; Priya and Blekhman 2019). The relative stability of an individual’s gut microbiome, once established, is imparted by the symbiotic relationships among the thousands of microbial species and the interaction of these microbial communities with the body (Kinross et al. 2011; Steinway et al. 2015).

The microbial diversity in the human colon is supported by a complex ecosystem composed of distinct microenvironments (Donaldson et al. 2016), created in part by longitudinal variations in nutrient availability, pH, and bile acids and radial gradients of signaling molecules, as well as immune factors, mucus, and antimicrobials produced by the gut epithelium (Ravcheev et al. 2019). Colonic oxygen also has a major impact on microbial geography in the gut (Albenberg et al. 2014; Espey 2013). Oxygen supplied to the colon is principally used by cells within the gut epithelium and the residual dissipates in a steep concentration gradient radially from the epithelial surface (Glover et al. 2016; Zheng et al. 2015). A surprisingly large fraction of gut bacteria, including many that have been previously thought to be strict anaerobes, have the capacity for aerobic respiration or mechanisms for survival under microaerobic conditions (Ravcheev and Thiele 2014). These bacteria reside in the epithelial mucosal layer and consume oxygen, which assists the survival of strict aerobes in the anoxic colonic lumen. Excess oxygen in the colon can disrupt the normal microflora, which has been hypothesized to contribute to inflammation and infiltration by pathogens (Rigottier-Gois 2013; Rivera-Chávez et al. 2017).

The conditions present in the gastrointestinal tract are difficult to accurately represent, but several *in* *vivo* and *in vitro* approaches have led toward improved understanding of human gut microbiome physiology. Animal models based on gnotobiotic mice accurately represent several aspects of the human gut microbiome but, due to their cost and low-throughput, are inadequate for screening applications. Platforms such as the Simulator of the Human Intestinal Microbial Ecosystem (SHIME) (Van de Wiele et al. 2015; Venema and Van den Abbeele 2013), PolyFermS (Fehlbaum et al. 2015), TIM-2 (Minekus et al. 1999), and others (Feria-Gervasio et al. 2014) use a parallel series of bioreactors to represent different gastrointestinal microenvironments. These systems have excellent process control for maintaining culture of a single human microbiome for days/weeks to enable in-depth studies over time (Marzorati et al. 2014), but are limited in their throughput and ability to examine variation between different donors. More simple single-batch fermentation systems like the Kobe University Human Intestinal Microbiome Model (KUHIMM) (Takagi et al. 2016), the miniature bioreactor platform (Auchtung et al. 2015), or commercially-available fermenter systems (for example the BioLector by m2p-labs, Baesweiler, Germany) can be multiplexed to culture more microbiomes (Wolfe 2018) but operate at a fixed oxygen environment, typically anaerobic. Both types of systems make use of a nutrient-rich medium feed to enable long-term culture, which significantly shifts the microbiome composition from that of the fecal initial fecal slurry (Li et al. 2018). Given the aforementioned limitations of these commercial platforms, we developed a 40-plex benchtop platform with a tunable oxygen gradient across each well that supports the short-term (24 h) maintenance of fecal microbiota with diverse oxygen requirements. This platform enabled testing the hypothesis that the presence of an oxygen gradient could help promote microbial interactions that are required to maintain the diversity and the relative abundance of the human gut microbiome in fecal donor samples. As a benchmark experiment, human fecal donor samples were used to demonstrate the improved preservation of the overall composition of the human microbiome compared to standard anaerobic culture as well as the ability to isolate gut microbes that occupy distinct oxic and anoxic microenvironments.

## Methods

### *In vitro* oxygen gradient platform set-up

**Fig. 1:**
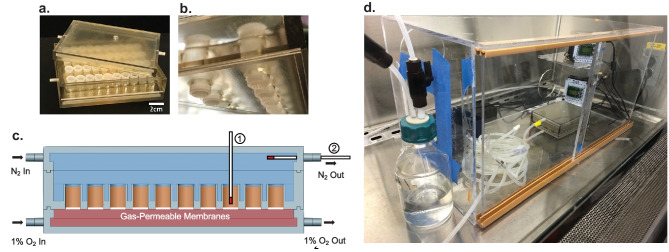
The 40-plex *in vitro* oxygen gradient platform. **a** Photograph of the 3D-printed platform containing 40 modified filter tube inserts for culture. **b** Photograph of the bottom of the culture layer showing the bottom of the filter tube inserts. **c** CAD cross-section of the system highlighting the 0% oxygen chamber (top; blue) and microoxygen chamber (bottom; red). The two optical oxygen probes, labeled 1 and 2, are inserted just off the bottom of a filter tube insert and the outflow of the 0% oxygen chamber respectively. **d** Complete setup including two convection heating fans for maintaining temperature in a custom Plexiglas box, gas humidification bubble jar, and tubing for gas delivery

3D-printing was used to produce the *in vitro* oxygenated platform to facilitate more rapid prototyping and scale-up compared to traditional hot embossing or injection molding techniques. The device is composed of a 40-chamber clamshell case with customized filter tube inserts (Fig. 1a-c). Designs were made in CAD software (Solidworks) and 3D-printed from a photocurable acrylated urethane resin (VeroClear, Stratasys) using a J750 PolyJet printer (Stratasys). Inserts consist of 0.75 mL centrifugal tubes that contain 5 µm filters (Millipore Ultrafree) impregnated with ~100 mg of polydimethylsiloxane (PDMS) (Dow Corning Sylgard 184) cured at 70 °C for 1 h to make the insert filters impermeable to liquid but not gas. The 3D-printed rack to hold the inserts has an array of tapered holes to create effectively gas-tight seals with the inserts. Inserts can be removed after an experiment and replaced, making the device reusable. Finally, chambers clamshelled around the rack create an assembled device with separate gas-tight compartments above and below the inserts with oxygen concentrations that can be tuned by the end user. Tubing interconnects printed on the outsides of the covers allow simple, rapid connection to gas lines to establish consistent, linear oxygen gradients across each insert and to enable operation outside of an anaerobic chamber.

The platform was operated in a cemented, laser-cut, optically-clear Plexiglas climate-controlled chamber (ePlastics.com) with two programmable digital convection heaters (incubator.com) (Fig. 1d). Gas flow (humidified 5% CO_2_/N_2_) was controlled by an ultralow rate gas flow (~50 mbar) controller (Fluigent). The gas feed was split via a y-connector into one line with gas-impermeable, polyvinylchloride (PVC) tubing (anaerobic feed to the top chamber) and one with four feet of oxygen-permeable Tygon tubing to allow ambient air to diffuse into the gas line (1% nominal), eliminating the need for a separate oxygen supply. The absolute oxygen concentration in the gas-permeable line can be tuned to within a few hundredths of a percent by adjusting the gas flow rate controller.

### *In vitro* oxygen gradient platform operation

An outline of the experimental protocol is shown in Supplementary Fig. 1. Human fecal material from three deidentified donors was obtained as FMT-R packs from OpenBiome (Cambridge, MA). Samples were thawed and diluted under anaerobic conditions to a ~10% slurry with sterile, reduced PBS containing 0.5% cysteine. No additional nutrients were added to enable oligotrophic culture. Prior to culture, triplicate samples (50 µl) were collected from each donor mixture (initial fecal slurry) and stored at -80 °C. Triplicate 0.75 mL aliquots from each initial fecal slurry were then cultured at 37 °C under an oxygen gradient in wells of the oxygen gradient platform or in tubes left in anaerobic conditions. After 24 h, single samples were collected from each anaerobic culture and three samples were collected from each well of the culture system: one each from the top and bottom quarter of each well prior to mixing, plus another collected after mixing the contents of the well by pipetting.

Samples were diluted and plated onto reduced BHI agar (Anaerobe Systems, Morgan Hill, CA). 16S rRNA analysis was performed with V4 515-806 primers, and sequence data derived from the sequencing process was processed using the MR DNA ribosomal and functional gene analysis pipeline (www.mrdnalab.com, MR DNA, Shallowater, TX). In summary, sequences were joined, depleted of barcodes then sequences with < 150 bp or with ambiguous base calls were removed. Sequences were denoised, chimeras removed, and operational taxonomic units (OTUs) defined by clustering at 3% divergence (97% similarity). Final OTUs were taxonomically classified using BLASTn against a curated database derived from RDPII and NCBI (www.ncbi.nlm.nih.gov, http://rdp.cme.msu.edu). OTUs were filtered for low prevalence (seen more than 3 times in 5% of samples) and low abundance (more than 10 total reads across all samples) and normalized using relative abundance (per sample OTU reads divided by the total reads per sample). Alpha and beta diversity were determined using the R phyloseq package [26], with unfiltered data was used for alpha diversity analysis to preserve species richness.

## Results

### Oxygen gradient equilibration and measurement

**Fig. 2:**
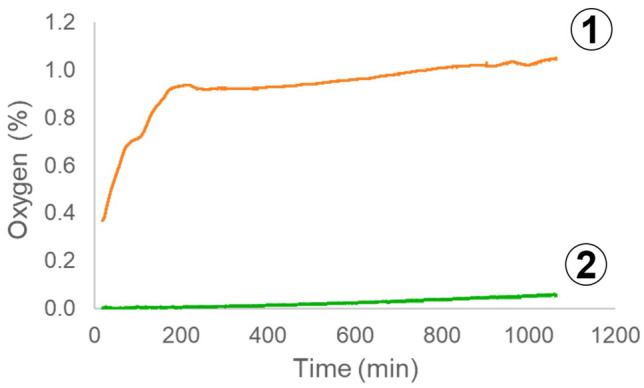
Oxygen traces of a PBS control sample in the *in vitro* oxygen gradient platform taken the bottom of a filter tube insert filled with reduced PBS (orange) and the 0% oxygen chamber (green) using fiber optic oxygen sensors. Numbers correspond to the position of the probe as indicated in Fig. 1c

To set up and calibrate the device prior to microbiome culture, the bottom gas flow was adjusted to 1% oxygen, with the top gas flow anaerobic (N_2_ only). Calibration of the 1% oxygen input was accomplished by inserting the fiber optic sensor directly into the gas stream via the outlet port and finely adjusting the gas ultralow flow regulator. In a well that contained reduced PBS, the oxygen gradient slowly develops over a period of approximately three hours, with the sensor positioned at the bottom of the well equilibrating at an oxygen level comparable to the bottom gas chamber (Fig. 2). The slow equilibration time is not unexpected, as the oxygen gradient across the well is driven by passive diffusion through the buffer. After equilibration, the gas gradient remained stable over an 18-h measurement period. The slow gradual increase in measured oxygen over the monitoring period (0.1 to 0.2% measured oxygen) observed with both sensors (well and the nitrogen output of the top chamber) is likely due to sensor drift, which has been previously observed with dye-based sensors at low oxygen concentrations (Bittig et al. 2018; Van Ganse et al. 2019). Maintenance of 1% input oxygen to the bottom of each chamber is expected to be more than sufficient to support growth via microaerobic respiration (Morris and Schmidt 2013). A principal limitation of the dye-based optical sensors is their inability to operate in opaque media like high density microbial cultures. As such, monitoring the oxygen gradient during active culture in the device was accomplished by loading a “blank” well in the 40-chamber device with reduced PBS that was monitored by the optical probe, with the remaining wells filled with microbiome samples.

### Comparison of human fecal microbiome culture under anaerobic conditions vs an applied oxygen gradient in the *in vitro* oxygen gradient platform

The initial microbial composition of the three donor microbiomes varied substantially. There was an average of 27,076 ± 854 reads per sample after removal of low abundance taxa. Members of the Bacteroidetes and Firmicutes phyla made up over 90% of the microbial constituents in each microbiome, but the ratio of Firmicutes/Bacteroidetes ratio was 3.3, 1.3, and 0.69 in donors A, B, and C respectively (Supplementary Table 1) indicating their compositional diversity. The microbial diversity (Shannon) of the initial fecal slurry was similar in the microbiomes of donors A and B (~4.0), but that of donor C (~3.4) was lower.

There was no significant difference in the bacterial concentration of samples from all three microbiomes before and after culture as determined by anaerobic plating onto BHI. The initial fecal slurries contained 1.7 × 10^9^, 2.0 × 10^9^, or 3.2 × 10^8^ cfu/ml for microbiomes A, B, or C, respectively. The A, B, or C samples after anaerobic culture contained 1.1 × 10^9^, 1.2 × 10^9^, or 2.1 × 10^8^ cfu/ml and after device culture contained 1.4 × 10^9^, 9.0 × 10^8^, or 4.1 × 10^8^ cfu/ml. This indicated that there was not a significant alteration in the total number of bacteria after 24-h culture.

**Fig. 3:**
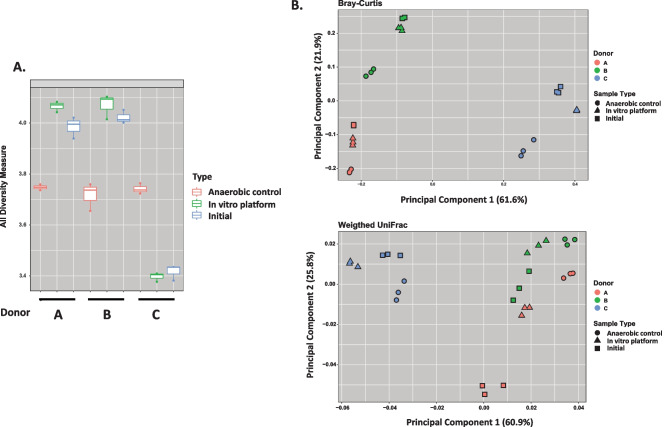
Comparison of initial microbial composition of human fecal microbiomes from three donors to that after 24-h oligotrophic incubation in the *in vitro* oxygen gradient platform or in anaerobic conditions. **a** Alpha-diversity (Shannon) index of OTUs identified in triplicate samples from the starting initial fecal slurry (blue) or after 24-h culture in the *in vitro* oxygen gradient platform device (green) or in anaerobic culture (red). The mean, standard deviation from the mean, and confidence intervals are indicated. **b** Beta-diversity comparison using principal component analysis (Bray-Curtis or Weighted UniFrac) of OTUs identified in triplicate samples from the starting initial fecal slurry (square) or after 24-h culture in the *in vitro* oxygen gradient platform (triangle) or in anaerobic culture (circle) of three human fecal microbiomes (indicated by color)

The human microbiomes maintained in an oxygen gradient in the *in vitro* oxygen gradient platform more closely resembled the pre-culture diversity and relative microbial abundance than those maintained under anaerobic conditions. For all three microbiomes, there was no significant difference in the Shannon (alpha) diversity between the initial fecal slurry and in samples maintained for 24 h in the *in vitro* oxygen gradient platform (Fig. 3a). In contrast, 24-h culture under anaerobic conditions altered the Shannon diversity, decreasing it in donor A and B microbiomes while increasing it donor C. Interestingly, after 24-h anaerobic culture, the resulting communities from all three donors had a similar Shannon diversity. Beta diversity, calculated using Bray–Curtis at the OTU level, indicated that the culture maintained in the *in vitro* oxygen gradient platform was also more similar in composition to the initial fecal slurry compared to a culture maintained under anaerobic conditions (Fig. 3b). This was evident with each of the three donors. This was also evidenced when the composition of the microbiomes was compared using weighted Unifrac analysis.

**Fig. 4:**
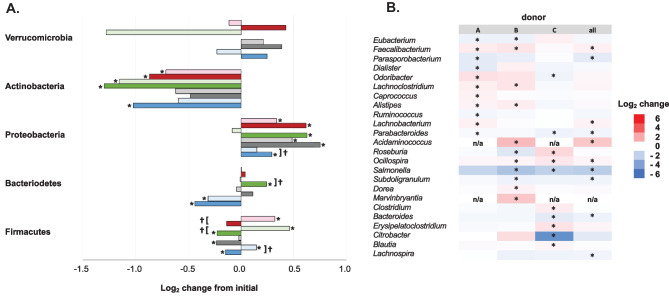
Changes in the microbial compositions of human fecal microbiomes from three donors cultured for 24-h in anaerobic conditions or in the *in vitro* oxygen gradient platform. **a** Log_2_ change in the average reads in triplicate samples from the initial fecal slurry after anaerobic (dark bars) or *in vitro* oxygen gradient platform (light bars). Data for the 5 most prevalent phyla are indicated for three different donor microbiomes (blue, gray, and green) and analysis of samples from three combined donors (red). A single asterisk (*) indicates a difference compared to initial fecal slurry with an adjusted p-value < 0.05. A dagger (†) indicates a difference between the anaerobic and *in vitro* oxygen gradient platform with an adjusted p-value < 0.05. **b** Log_2_ difference in average reads at the genus level in triplicate samples of human gut microbiome cultured for 24-h under anaerobic conditions compared to the *in vitro* oxygen gradient platform. Data from three human donors and combined samples is indicated, with those with an adjusted p-value < 0.05 indicated (*). Only those genera with a significant difference in one donor or combined samples are indicated. Genera not found in a particular sample are displayed (n/a)

The microbial taxa that were enriched or depleted in abundance after 24-h culture, identified using DEseq2 analysis (Love et al. 2014), in the *in vitro* oxygen gradient platform or under anaerobic conditions when compared to the initial fecal slurry varied by the microbiome donor. All three donor microbiomes showed a small but significant decrease in the abundance of Firmicutes from the initial fecal slurry after anaerobic culture (Fig. 4a). This change was not evident after culture in the *in vitro* oxygen gradient platform, with the abundance of Firmicutes significantly increased relative to the initial fecal slurry with two donor fecal microbiomes (donors A and C). Shifts in the prevalence of Bacteroidetes, the other major fecal microbiome constituent, were more donor-dependent. The fecal microbiome of donor A, which had a lower abundance of Bacteroidetes than the other microbiomes, showed a significant decrease in that phylum’s abundance after both anaerobic and oxygen gradient incubation. The abundance of Proteobacteria significantly increased with all three fecal microbiome donors after anaerobic incubation, and after oxygen gradient exposure with one donor. With all donors, there was a lower abundance of Actinobacteria in samples from both culture conditions (*in vitro* oxygen gradient platform and anaerobic) compared to the initial fecal slurry (Fig. 4a).

When the average microbial abundance from paired anaerobic and oxygen gradient samples from each donor were compared, the abundance of Firmicutes was significantly decreased after anaerobic culture compared to culture in the *in vitro* oxygen gradient platform with two donors. In addition, one donor culture (donor C) had a significant difference in the prevalence of Bacteroidetes after culture in the two conditions.

At the genus level, there were 24 genera whose average abundance differed significantly (adjusted p-value < 0.05) in at least one fecal microbiome culture after 24-h under anaerobic conditions compared to the *in vitro* oxygen gradient platform (Fig. 4b). *Salmonella* and *Citrobacter*, two facultative aerobes, showed significantly lower abundance after anaerobic culture compared to after oxygen gradient incubation while the anaerobic Firmicutes *Faecalbacterium*, *Alistipes*, *Lachnoclostridium*, and *Acidaminococcus* had significantly increased abundance after anaerobic incubation. Notable was the number of genera that showed significantly different abundance in the two conditions that were specific to the fecal microbiome culture from one donor (Supplementary Fig. 2).

### Comparison of human fecal microbiome culture in regions of the *in vitro* oxygen gradient platform with different oxygen content

**Fig. 5:**
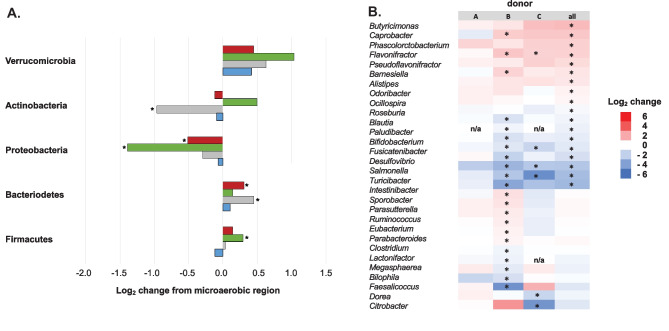
Differences in the microbial compositions of samples of human fecal microbiome culture taken from the anaerobic (top) and microaerobic (bottom) regions of the *in vitro* oxygen gradient platform after 24-h. **a** Log_2_ change in the average reads of triplicate samples from the anaerobic (top) sample compared to the microaerobic (bottom) sample. Data for the 5 most prevalent phyla are indicated for three different donor microbiomes (blue, gray, and green) and the combined samples (red). An asterisk (*) indicates a difference with an adjusted p-value < 0.05. **b** Log_2_ difference in average reads in triplicate human gut microbiome culture samples between the anaerobic (top) and microaerobic (bottom) regions of the *in vitro* oxygen gradient platform. Data from three human donors and combined samples is indicated, with those with an adjusted p-value < 0.05 indicated (*). Only those genera with a significant difference in one donor or combined samples are indicated. Genera not found in a particular sample are displayed as (n/a)

We also compared the microbial composition of human fecal microbiome culture in samples taken from the anaerobic (top – closer to N_2_ input) and microaerobic (bottom – closer to O_2_ input) of wells in the *in vitro* oxygen gradient platform to determine if different microenvironments were generated. Significant differences in the microbial composition were observed, with the taxa associated with these changes specific to the individual human fecal microbiomes (Fig. 5). The donor A microbiome culture displayed no significant differences in the average microbial composition of the *in vitro* oxygen gradient platform anaerobic (top) and microaerobic (bottom) samples at the phylum or genus levels. In contrast, donor B showed a significant relative increase in the prevalence of Bacteroidetes and a decrease in the presence of Actinobacteria in the *in vitro* oxygen gradient platform anaerobic sample, as well as a significant increase in the abundance of 9 anaerobic genera and a decrease in the abundance of 12 genera, including aerobes like *Salmonella*, but also several anaerobes. Samples of the donor C fecal microbiome from the anaerobic region showed a significantly lower abundance of Proteobacteria and alterations to 11 genera including significant decreases in the abundance of *Salmonella* and *Citrobacter*, two facultative aerobes.

## Discussion

Compared to environmental microbial communities, the composition of the human gastrointestinal tract microbiome has little variation at the phylum level. The human gut is dominated by Bacteroidetes and Firmicutes, with the remainder mostly consisting of members of Proteobacteria, Actinobacteria, and Verrucomicrobia (Huttenhower et al. 2012; Qin et al. 2010). However, there is a remarkable diversity and variability among individuals at lower taxonomic levels (species and strain, for instance) (Lozupone et al. 2012). While much of the understanding of the gut microbiome has relied on metagenomics, comparative study of microbiome function *in vitro* can augment that knowledge. To accommodate that, we have developed a high-throughput culture system with an oxygen gradient to sustain microbial diversity and allows simultaneous study of multiple human fecal microbiome cultures. The system was designed to be simple to construct and set-up, and can be operated outside of an anaerobic chamber. We demonstrated that, within the parameters of our study, human gut microbiome culture in the *in vitro* oxygen gradient platform with an oxygen gradient better maintained the original microbial diversity and composition of a human fecal sample than standard culture under anaerobic conditions. This observation was consistent with three different human fecal microbiomes with distinct starting compositions. Particularly notable was the significantly lower abundance of Firmicutes in the anaerobic culture compared to that of the *in vitro* oxygen gradient platform with all three human fecal microbiomes. Since the vast majority of Firmicutes are considered strict anaerobes, this was unexpected. Twenty of the 28 genera with significant differences between the anaerobic *in vitro* oxygen gradient platform cultures belonged to Firmicutes, though the particular genera that varied largely depended on the donor. These shifts in microbial composition highlight the complex symbiotic relationship between major classes of gut bacteria, which contribute to gut homeostasis in many ways, from maintaining the redox potential to digesting complex carbohydrates (Flint et al. 2012).

The *in vitro* oxygen gradient platform has the capability to explore the effects of different experimental parameters on human fecal microbiome culture. We chose oligotrophic culture conditions for the initial study since they were shown, in the short term, to maintain microbial composition of a human microbiome culture compared to culture in rich medium (Long et al. 2015). Maintaining the composition of human fecal microbiome culture for longer than 24 h would require a continuous nutrient supply via a medium feed, a feature that is currently being implemented to the *in vitro* oxygen gradient platform. Oxygen supply is another adjustable experimental parameter. With input of 1% oxygen, differences in the microbial composition in the *in vitro* oxygen gradient platform compared to anaerobic culture and in the top (anaerobic) and bottom (microaerobic) microenvironments of the *in vitro* oxygen gradient platform wells were evident. However, the oxygen levels during bacterial culture could not be measured due to the nature of the oxygen probe, and it is likely that a steeper oxygen gradient than that measured in PBS was present due to bacterial oxygen consumption. Additional experiments using the *in vitro* oxygen gradient platform with different amounts of input oxygen, which is easily adjustable, would enable correlation of the oxygen gradient depth to the effects on the human fecal microbiome culture composition.

The simplicity of our *in vitro* oxygen gradient platform and its ability to multiplex differentiates it from other reactor or fermentation systems used for study of the human gastrointestinal tract (Payne et al. 2012). In the *in vitro* oxygen gradient platform, microenvironments that recreate natural linear gradients of other factors that affect microbial metabolism such as pH, hydration, or osmolality, could be generated by changing the conditions in different wells. In addition, antimicrobials, immune factors, or mucus, could be added to the microaerobic (bottom) environment to mimic conditions in proximity of the colonic epithelium (Jalili-Firoozinezhad et al. 2019). The platform format is compatible with serial sample collection for genomic, transcriptomic, or metabolomic studies of microbiome function and response. It also enables simultaneous study of the anaerobic and microaerobic microbial communities from the same human microbiome sample. These unique capabilities will help advance the understanding of the bacterial interactions in the human gut microbiome.

## Supplementary Information

Below is the link to the electronic supplementary material.

Supplementary file1 (PDF 708 KB)

## Data Availability

The datasets generated during and/or analyzed during the current study are available from the corresponding author on reasonable request.

## Declarations

### Distribution statement

A. Approved for public release. Distribution is unlimited. © 2022 Massachusetts Institute of Technology. Delivered to the U.S. Government with Unlimited Rights, as defined in DFARS Part 252.227-7013 or 7014 (Feb 2014). Notwithstanding any copyright notice, U.S. Government rights in this work are defined by DFARS 252.227-7013 or DFARS 252.227-7014 as detailed above. Use of this work other than as specifically authorized by the U.S. Government may violate any copyrights that exist in this work.
