# Supplementary material for: An in vitro platform for study of the human gut microbiome under an oxygen gradient
Source: Biomed Microdevices. 2023 Apr 4;25(2):14. doi: 10.1007/s10544-023-00653-3 (PMC10073063; doi:10.1007/s10544-023-00653-3)
Supplement: Supplementary file 1 — Supplementary file1 (PDF 708 KB) [file 10544_2023_653_MOESM1_ESM.pdf]

An In Vitro Platform for Study of the Human Gut Microbiome under an Oxygen Gradient

James Comolli[a], David I. Walsh III[a], Johanna Bobrow[a], Chelsea Lennartz[a], Nicholas J. Guido[a], Todd Thorsen[a*]

a. Biological & Chemical Technologies, MIT Lincoln Laboratory, Lexington, MA, USA

*Corresponding author
Todd Thorsen
Email: thorsen@ll.mit.edu

SUPPLEMENTARY INFORMATION

Supplemental Table 1. Relative abundance of phyla in human fecal microbiomes from different donors prior to culture.

	Donor A	Donor B	Donor C
Firmicutes	72.41 ± 0.92	54 ± 0.71	39.17 ± 1.66
Bacteroidetes	22.13 ± 0.58	41.27 ± 0.86	56.98 ± 1.32
Proteobacteria	5.07 ± 0.14	4.37 ± 0.21	3.64 ± 0.17
Verrucomicrobia	0.22 ± 0.02	0.01 ± 0	0 ± 0
Actinobacteria	0.08 ± 0.01	0.33 ± 0.08	0.21 ± 0.03
Spirochaetes	0.08 ± 0.01	0.02 ± 0.01	0 ± 0
Others	0.01 ± 0	0 ± 0	0 ± 0
Firmicutes/Bacteroidetes ratio	3.27	1.31	0.69

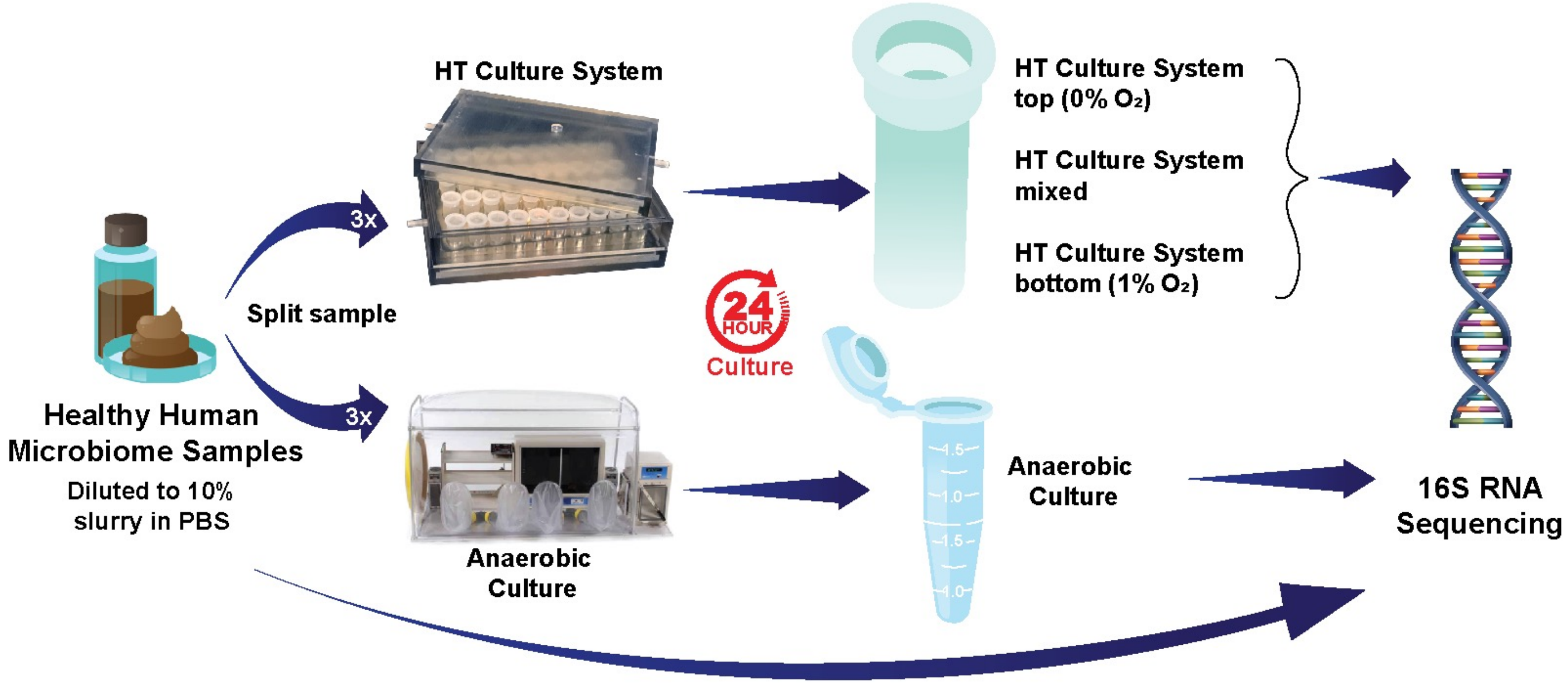

Supplemental Figure 1. Experimental protocol for human fecal microbiome study using the in vitro oxygen gradient platform

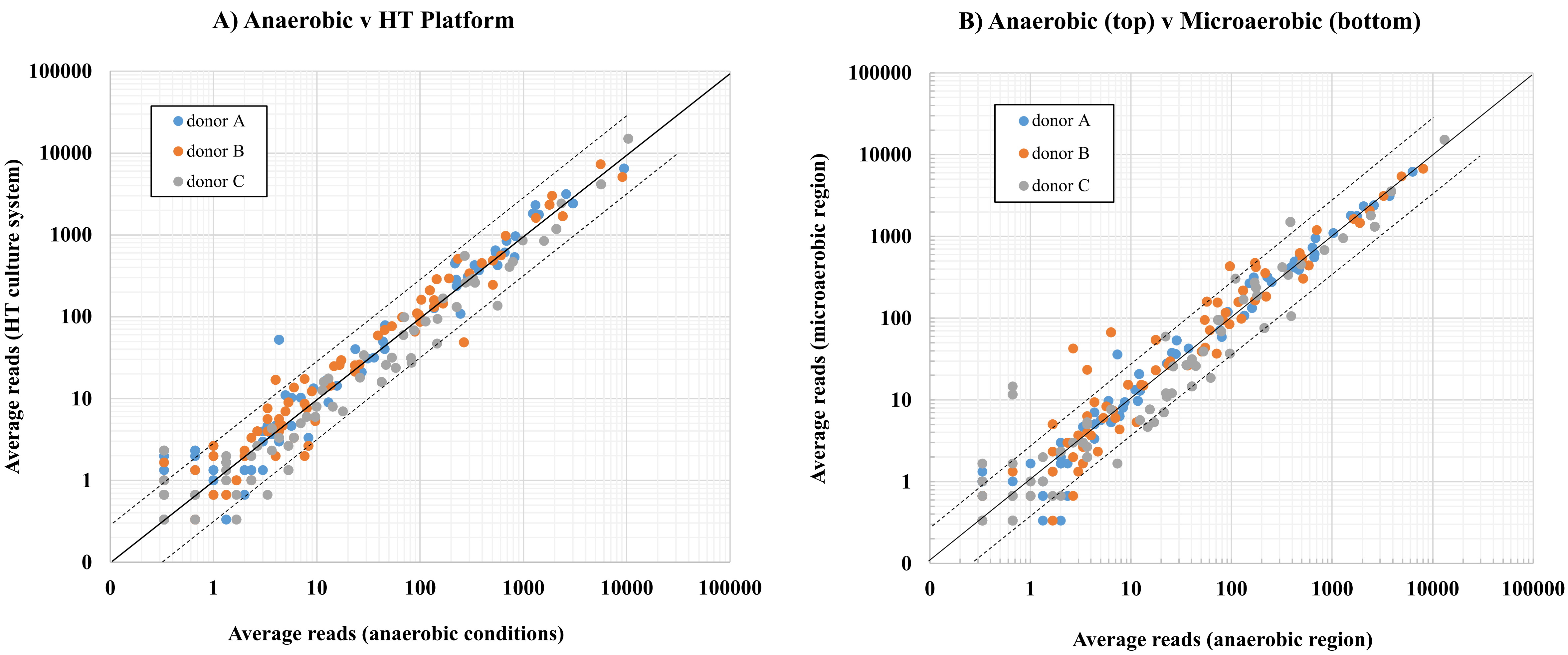

Supplemental Figure 2. Average reads assigned to genera in fecal microbiomes from donor A (blue), donor B (orange), or donor C (gray) after culture for 24 hours comparing A) anaerobic conditions compared to the in vitro platform or B) anaerobic and microaerobic regions from chambers within the in vitro platform. The solid line indicates a linear relationship between the conditions while the dotted lines represent a 2-fold increase or decrease.
